# Zika genomics urgently need standardized and curated reference sequences

**DOI:** 10.1371/journal.ppat.1006528

**Published:** 2017-09-07

**Authors:** Kristof Theys, Pieter Libin, Kai Dallmeier, Andrea-Clemencia Pineda-Peña, Anne-Mieke Vandamme, Lize Cuypers, Ana B. Abecasis

**Affiliations:** 1 KU Leuven – University of Leuven, Department of Microbiology and Immunology, Rega Institute for Medical Research, Clinical and Epidemiological Virology, Leuven, Belgium; 2 Artificial Intelligence Lab, Department of computer science, Vrije Universiteit Brussel, Brussels, Belgium; 3 KU Leuven – University of Leuven, Department of Microbiology and Immunology, Rega Institute for Medical Research, Laboratory of Virology and Chemotherapy, Leuven, Belgium; 4 Molecular Biology and Immunology Department, Fundación Instituto de Immunología de Colombia (FIDIC), Basic Sciences Department, Universidad del Rosario, Bogotá, Colombia; 5 Global Health and Tropical Medicine, GHTM, Institute for Hygiene and Tropical Medicine, IHMT, University Nova de Lisboa, UNL, Lisbon, Portugal; University of Pittsburgh, UNITED STATES

Emerging viral epidemics pose a threat to public health globally. The recent spread of the Zika virus (ZIKV) across the Pacific region and the Americas is particularly disturbing, given its association with severe birth malformations. On February 1, 2016, the World Health Organization (WHO) declared ZIKV a Public Health Emergency of International Concern, which prompted a global response to improve our understanding of ZIKV epidemiology and disease manifestations. Intensified ZIKV surveillance and real-time sharing of novel scientific evidence coincided with a scaling up of ZIKV whole genome sequencing for public health purposes and diagnostics [1,2]. As a result, most human ZIKV genomes available to date originate from the ongoing outbreak. While this proliferation of genomic data offers new opportunities for comparative and evolutionary genomics of ZIKV, we demonstrate that the rapid advance in ZIKV genomics has resulted in inconsistencies that complicate the interpretation, reproducibility, and comparison of findings from and across studies, particularly due to the lack of consensus on a standardized and representative reference genome annotation. ZIKV reference genomes do not match virus strains sampled from the global epidemic or are not adequately annotated at the protein level, and current heterogeneity in study methodology ultimately limits the full potential of ZIKV genomics. In this letter, we address the need for curation and standardized annotation of ZIKV reference genomes in order to guide researchers and clinicians in genomic analyses and the translation of research findings.

**Fig 1:**
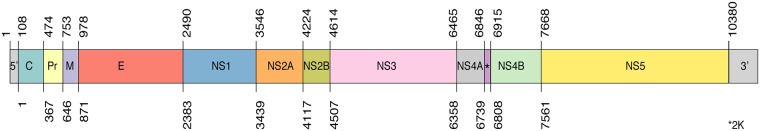
Visual representation of the correct genome annotation for the proposed ZIKV reference sequence KJ776791 [10]. The figure shows the corrected nucleotide positions for the Genbank annotation of KJ776791. This correction was guided by the curated annotation of NC_012532 [4]. The upper axis indicates nucleotide positions of proteins in the full-length genome (10,807 nt), including the 5’UTR (length of 107 nt) and 3’UTR (length of 428 nt). The lower axis indicates nucleotide positions of encoded proteins in the CDS (amino acids: 1–3,423; nucleotides: 1–10,269). These positions also apply as the correct annotation of the WHO candidate reference genome KX369547. While the reference sequence we propose encompasses the complete UTR, we note that the lengths of the 5’UTR and 3’UTR of the WHO candidate reference sequence KX369547 are limited to 90 nt and 406 nt, respectively.

Reference genomes are used as templates for a wide range of sequence-based applications and should be carefully selected to represent pathogen diversity from a temporal, geographical, and epidemiological perspective. Kuno and Chang (2007) reported the first ZIKV genome with a detailed description and annotation of the coding sequence (CDS) (Genbank accession number [GAN] AY632535). This sequence was based on the MR-766 strain that was isolated in 1947 from a sentinel rhesus monkey at the Zika forest in Uganda [3]. The National Center for Biotechnology Information (NCBI) released their reference sequence for ZIKV in 2016, using the AY632535 sequence to guide the annotation of the ZIKV reference genome (GAN NC_012532) [4]. With a CDS length of 3,419 amino acids (aa), AY632535 and NC_012532 lack a 4-aa N-glycosylation motif (VNDT) in the envelope region that is present in all human ZIKV genomes sampled during the recent global epidemic, including the candidate reference strain (GAN KX369547) proposed in 2016 for quantification of ZIKV RNA on behalf of the WHO [1] (Fig 1). This loss has been attributed to an artifact of repeated serial passages or adaptation to a mosquito vector, while in vivo restoration was observed upon replication in macaques, and the motif seems to play a role for viral particle structure and transmission [5,6,7,8]. The origin and biological role of the deletion is not yet resolved, and site alterations can be seen in both historical and contemporary ZIKV genomes, with 4-aa or 6-aa deletions and a T-to-I substitution, even between different MR-766-derived sequences [5,7,8]. A direct implication of this discrepancy in genome lengths is that the motif is ignored in downstream analyses and that comparative studies report discordant genomic coordinates for a similar observation. The ZIKV reference genome must include the 4-aa motif and be representative for the virus population circulating in the human population. As such, past and future genomic analyses should be updated to harmonize findings. While the WHO reference genome meets these criteria, its sequence (10,769 nt in length) does not cover the full-length ZIKV genome (10,807 nt in length) due to partial 5’ and 3’ untranslated regions (UTRs) (Fig 1).

More importantly, the reference genome of choice needs to be well annotated to allow consistent identification of genomic regions when comparing viral strains. NCBI staff has manually curated the ZIKV genome sequence reported by Kuno and Chang [3,4] using the predicted protease cleavage site information from the original study. The value of this validation effort is significant because it corrected the protein lengths reported by Kuno and Chang (Table 1). However, a similar curation by NCBI of ZIKV genomes that contain the 4-aa motif is lacking, and this results in considerable heterogeneity in reported annotation of ZIKV peptides that can be observed in recent literature and public databases. Firstly, MR-766 genomes with a CDS length of 3,423 aa have been submitted to Genbank without a complete and correct annotation, reducing their value as a reference sequence. For example, the nonhuman GAN KX377335 strain has served as guiding sequence in recent molecular epidemiology studies, but genomic sequences originating from these studies (e.g., GAN KU940224) were submitted to Genbank with a protein annotation that differs significantly from the curated NCBI reference genome [2,9] (Table 1). Secondly, the WHO candidate reference strain KX369547 has a complete protein annotation in Genbank, but protein lengths differ markedly from the NCBI reference genome (Table 1), which affects its current use as reference. Strikingly, protein annotations of ZIKV genomes submitted by studies using KX377335 as the reference sequence propagate the incorrect protein annotations of the human candidate WHO strain (Table 1). Thirdly, studies have reported ZIKV protein lengths of human strains (GAN KJ776791) that are inconsistent with any of the annotations discussed above, causing more confusion with respect to the genome structure of ZIKV [10]. Fourthly, ambiguous UTR lengths are found in ZIKV genomes available in Genbank, without being labeled as partial UTR.

**Table 1:** High level of heterogeneity in reported peptide lengths of ZIKV from genome annotations available in Genbank or protein information reported in original publications.

**Genbank accession number**	AY632535 [3]	NC_012532 [4]	KX377335 [9]	KU940224 [2]	KX369547 [1]	KJ776791 [10]
**Genbank annotation available**	No	Yes	No	Yes	Yes	Yes
**Source of protein information**	Original publication	Genbank	Original publication	Genbank	Genbank	Original publication
**Additional information**	Sequence derived from MR-766 strain	Curated AY632535 by NCBI	Sequence derived from MR-766 strain	Sequence aligned with KX377335	WHO reference strain	Annotation differs from publication (published pre-2015)
**Source**	Monkey	Monkey	Monkey	Human	Human	Human
**CDS length (in aa)**	3,419	3,419	3,423	3,407	3,423	3,424
**C**	122	122	122	109	125	105
**prM**	178	168	168	165	165	187
**E**	500	500	504	505	505	505
**NS1**	342	352	352	362	362	352
**NS2A**	226	226	226	218	218	217
**NS2B**	130	130	130	144	144	139
**NS3**	617	617	617	603	603	619
**NS4A**	127	127	127	147	147	127
**2K**	23	23	23	-	-	-
**NS4B**	252	251	251	502	502	255
**NS5**	902	903	903	652	652	904

As for any pathogen, an integrated map of ZIKV genomewide diversity and genetic processes that drive the tempo and mode of evolution advances the design of diagnostics, therapeutics, vaccines, and other control strategies. However, an adequate framework for ensuring standardized genome analysis methods and curation of (meta-)data is lacking for ZIKV. The NCBI Reference Sequence Database is widely used as the primary reference for genomics. While multiple sources in the public domain make the efforts to annotate and curate viral genomes adequately, these sources are not yet widely and routinely used. Clinicians and researchers can be misinformed by information submitted to Genbank, and the erroneous translation of study findings can challenge the advance of ZIKV knowledge. Therefore, it is pivotal that a single reference Genbank entry for ZIKV is created with a CDS length of 3,423 aa, complete UTR regions, and a correctly curated genome annotation. Despite its careful selection criteria, we do not support the human WHO candidate reference strain KX369547 as preferred reference sequence for comparative genomics purposes [1]. Instead, we propose the use of the ZIKV genome sequence GAN KJ776791 [10]. This sequence matches well with temporal, geographical, and epidemiological characteristics of the WHO reference strain, but in addition, KJ776791 has complete UTRs (Fig 1). The CDSs of KX369547 and KJ776791 are genetically similar, differing only at 8 nucleotide positions and 2 amino acid positions. The widespread distribution of the amino acid substitutions (L42F in the M protein and E146K in the NS1 protein) in the recent epidemic further support the use of KJ776791 over KX369547 (http://www.nextstrain.org/zika). To this end, the protein annotation of KJ776791 (and of KX369547) needs to be corrected using predicted consensus protease cleavage sites found in the ZIKV open reading frame or, ideally, supported by experimental data [3,4]. Fig 1 gives a visual representation of our proposed annotation of KJ776791 and of the current WHO reference strain. Detailed information on this corrected annotation can be found at http://rega.kuleuven.be/cev/reference-sequences/rega-zikv. Furthermore, the selection of additional reliable and curated reference sequences is warranted to overcome current inconsistencies and to establish a set representative for worldwide ZIKV genetic diversity. The scientific community should support such efforts, for example, by using data from the European Virus Archive (https://www.european-virus-archive.com).

As time passes, errors propagate in literature and become increasingly difficult to correct. Also, ZIKV genomic data, regardless of any reference potential, should be well curated and given sufficient metadata when made publicly available. Complete ZIKV genomes must cover UTRs and should be indicated accordingly when, in fact, limited to the complete CDS (with partial UTRs) or containing large regions of undetermined nucleotides. In fact, this lack of curation and standardized annotation impairs the accurate use of public data. In conclusion, electronic records can be adapted quickly, but tools for automated curation and verification of sequence data need to be implemented routinely and in a widespread manner. Researchers should also be more vigilant regarding annotation discrepancies.
